# Machine Learning for Predicting Postoperative Complications After Hypospadias Surgery: A 10-Year Single-Center Retrospective Cohort Study

**DOI:** 10.3390/children13070962

**Published:** 2026-07-21

**Authors:** Ling Li, Haosen Shen, Ying Qiu, Baoling Bai, Kexin Zhang, Shuangshuang Yang, Chen Shen, Jiaxin Cheng, Qin Zhang, Xianghui Xie

**Affiliations:** 1 Capital Institute of Pediatrics, Chinese Academy of Medical Sciences & Peking Union Medical College, Beijing 100730, China; 2 Department of Urology, Capital Center for Children’s Health, Capital Medical University, Capital Institute of Pediatrics, Beijing 100020, China; 3 Beijing Municipal Key Laboratory of Child Development and Nutriomics, Capital Center for Children’s Health, Capital Medical University, Capital Institute of Pediatrics, Beijing 100020, China; 4 Department of Urology, Beijing Friendship Hospital, Capital Medical University, Beijing 100050, China

**Keywords:** hypospadias, urethroplasty, postoperative complications, machine learning, prediction model

## Abstract

**Highlights:**

**What are the main findings?**

- An interpretable SVM-based prediction model for postoperative complications after hypospadias repair was developed and internally validated.
- LASSO retained four predictors: hypospadias type, surgical technique, surgeon experience, and patient age.
- SHAP analysis identified surgical technique as the most influential feature, followed by surgeon volume and hypospadias type.

**What are the implications of the main findings?**

- The model provides a clinically interpretable risk-stratification tool using routinely available variables, with SHAP offering visual insights into risk-associated factors.
- The comparable performance between SVM and LightGBM suggests that algorithm selection may have limited impact on predictive accuracy.
- External validation in multicenter cohorts is required before clinical implementation, and findings should be interpreted as risk-associated patterns rather than causal relationships.

**Abstract:**

**Objectives:** Hypospadias is one of the most common congenital malformations of the male genitourinary system, and postoperative complications remain a major concern affecting surgical outcomes and patients‘ quality of life. Whether machine learning models can effectively predict complication risk using routinely available clinical variables remains unclear. **Methods:** A retrospective analysis was performed on 671 hypospadias patients who underwent urethroplasty at the Department of Urology, Capital Children’s Medical Center, between December 2015 and September 2024. The final dataset included 671 patients (training set: 536; validation set: 135). The median follow-up duration was 48 months (range: 19 to 72 months). Least absolute shrinkage and selection operator (LASSO) regression with nested cross-validation within the training set was used for feature selection, followed by the development of five machine learning models (Random Forest, XGBoost, LightGBM, Logistic Regression, and Support Vector Machine). Model performance was evaluated using AUC, calibration curves, Brier score, and decision curve analysis. Feature importance was assessed using SHapley Additive exPlanations (SHAP). **Results:** LASSO retained four features for model development: hypospadias type, surgical technique, surgeon experience, and patient age. The overall complication rate was 22.9% (154/671). Among the models evaluated, the Support Vector Machine (SVM) showed the most balanced performance in the validation set, achieving an AUC of 0.810 and a Brier score of 0.157. LightGBM demonstrated comparable performance (AUC: 0.802). SHAP analysis identified surgical technique as the most influential predictor, followed by surgeon volume and hypospadias type, though these findings should be interpreted with caution given the confounding between surgical complexity and disease severity. **Conclusions:** An interpretable SVM-based prediction model was developed and internally validated to stratify risk for postoperative complications after hypospadias repair using routinely available clinical variables. SHAP provided clinicians with visual insights into key risk-associated factors. However, given the single-center retrospective design and lack of external validation, further multicenter prospective studies are warranted to confirm the generalizability of these findings before clinical implementation.

## 1. Introduction

Hypospadias is a relatively common congenital urethral abnormality, affecting approximately 0.69 per 10,000 live births in Asia, with the global prevalence increasing over time [1]. It results from incomplete closure of the urogenital groove during embryonic development and is clinically characterized by penile curvature, an abnormally positioned urethral meatus, and irregular foreskin distribution [2]. Approximately 50% of cases are distal, 30% midshaft, and 20% proximal [3]. Surgical reconstruction remains the mainstay of treatment, yet postoperative morbidity remains substantial despite refinements in technique [4]. Urethral fistula occurs in 4–28% of cases, meatal stenosis in up to 5.7%, and urethral stricture in 6–12% [5]. These events often necessitate reoperation and prolong follow-up [6]. The optimal surgical approach and timing continue to be debated, and no universally accepted standard has been established [7].

Recent nomogram-based models have been developed to predict complications. Mao et al. reported a nomogram (AUC 0.821) using six variables including glans width and urethral defect length [8]; Fang et al. identified three anatomical predictors in a multicenter TIP cohort (AUC 0.723) [9]; and Dong et al. developed a perididymis-covering nomogram (AUC 0.909) [10]. However, these models rely on measurements not uniformly recorded, and most use logistic regression, leaving machine learning underexplored.

Machine learning accommodates nonlinear interactions and has been applied to surgical outcome prediction [11,12], but its limited interpretability has been partially addressed by SHapley Additive exPlanations (SHAP) [13]. In this study, we used five ML algorithms on a cohort of 671 patients, incorporating only four routinely available predictors (hypospadias type, surgical technique, surgeon volume, and age) to develop an interpretable, internally validated risk model. Performance was evaluated using AUC, calibration metrics, and decision curve analysis [14,15], with SHAP enhancing clinical interpretability [13].

## 2. Materials and Methods

### 2.1. Patients

A total of 671 patients with hypospadias who underwent urethroplasty or glanuloplasty (e.g., MAGPI) at the Department of Urology, Capital Children’s Medical Center, Capital Medical University, Beijing, China, from December 2015 to September 2024 were enrolled in this study. Exclusion criteria were: (1) missing key clinical data (hypospadias type, surgical technique, surgeon, or follow-up outcomes); (2) loss to follow-up; (3) follow-up duration < 6 months; and (4) concurrent urological anomalies or prior urethral surgery. This study was approved by the Institutional Ethics Committee of the Capital Children’s Medical Center (Approval Number: SHERLL2023038), with informed consent waived due to the retrospective nature of the study. All procedures complied with the Declaration of Helsinki and confidentiality principles.

### 2.2. Follow-Up

**Figure 1:**
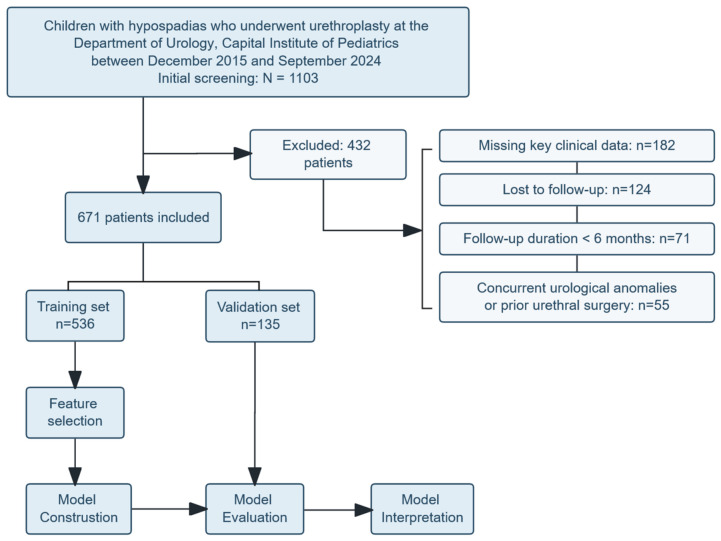
Flowchart of patient selection and model development.

Follow-up was conducted according to standard institutional protocols. The follow-up duration was defined as the period from postoperative discharge until the final follow-up examination. Patients attended outpatient examinations at 1, 3, 6, and 12 months postoperatively. Assessments included physical examination of the surgical site, evaluation for urethral fistula, and uroflowmetry in toilet-trained children. Parents were instructed to report recurrence-related symptoms such as urethral fistula or dysuria at the operative site. Suspected recurrence required further clinical evaluation, and recurrence dates were recorded by the attending physician based on clinical diagnosis (Figure 1).

### 2.3. Data Processing

Using a fixed random seed (42), patients were randomly assigned to the training (*n* = 536) and validation (*n* = 135) sets in an 8:2 ratio stratified by complication status, and no imputation was performed. The outcome variable was postoperative complications, defined as a composite endpoint including urethral fistula, urethral stricture, urethral diverticulum, and surgical-site infection. Complications were counted at the patient level, meaning each patient was counted once in the overall rate regardless of the number of complication types they developed; patients with multiple complications were recorded separately in each corresponding category, so the sum of individual counts may exceed the total number of patients. This definition and counting method were applied consistently across all models.

The final variables included age, BMI, hypospadias classification (coronal, penile, scrotal, and perineal), surgical techniques (MAGPI (Meatal Advancement and Glanuloplasty Incorporated), Mathieu (perimeatal-based flap urethroplasty), TIP (tubularized incised plate urethroplasty), Onlay (onlay island flap urethroplasty), Duckett (transverse preputial island flap urethroplasty), Koyanagi (parameatal-based flap urethroplasty), combined Duckett and Duplay, and two-stage repair), surgeon experience, and surgical period. Surgeon experience was defined as the cumulative number of hypospadias procedures performed by each surgeon during the study period, categorized as low-volume (≤200 cases) or high-volume (>200 cases) based on the median of the distribution of surgical volume among all participating surgeons, and surgical period was classified as early (2015–2019) or recent (2020–2024). Before model construction, continuous variables (age and BMI) were retained with their original values; unordered multi-category variables (surgical technique and hypospadias classification) were one-hot encoded, expanding to 18 binary features; and two-category variables (surgeon group and surgical period) were included directly. Variables such as glans width, urethral plate characteristics, degree of chordee, and preoperative hormonal therapy were not included in the analysis because they were not consistently recorded in the electronic medical records during the early study period (2015–2019). These variables were therefore unavailable for retrospective extraction, which we acknowledge as a limitation of the study.

### 2.4. Statistical Analysis

Continuous variables with normal distributions were compared using *t*-tests, reported as mean ± standard deviation. Non-normally distributed continuous variables were compared using the Mann–Whitney U test, reported as median (interquartile range [IQR]). Categorical variables are expressed as percentages and compared using Pearson’s chi-square (χ^2^) test, with categorical variables represented by counts and percentages. All analyses were performed using Python (version 3.9.21) and IBM Statistical Package for the Social Sciences software (version 27.0). Statistical significance was set at *p* < 0.05 (two-sided).

### 2.5. Feature Selection Process

Least absolute shrinkage and selection operator (LASSO) regression was applied for feature selection, with the optimal regularization parameter λ determined via nested 10-fold cross-validation within the training set using the one-standard-error rule. Given the limited number of complication events (*n* = 154) and candidate variables (*n* = 6), LASSO was used primarily for dimensionality reduction to mitigate overfitting and control multicollinearity. The selected features were locked for subsequent model development and were used for prediction purposes only, not for causal inference.

### 2.6. Model Development and Evaluation

Five supervised ML models were constructed and evaluated: Random Forest (RF), XGBoost, LightGBM, Logistic Regression (LR), and Support Vector Machine (SVM). Model development was conducted on the training set using 10-fold cross-validation. All models were implemented using default hyperparameters from the Python Scikit-learn library (version 1.2.1) to ensure standardized comparison across algorithms. This approach may have constrained model performance, and the results should therefore be interpreted in the context of these default settings. Hyperparameter optimization will be addressed in future studies. Taking the SVM as an example, the key parameter configurations are detailed as follows: the model was implemented with a radial basis function (RBF) kernel, with the regularization parameter C and kernel coefficient gamma set to default values (C = 1.0, gamma = ‘scale’), and class weight set to ‘balanced’ to address class imbalance. Predicted probabilities were generated using Platt scaling (default in scikit-learn’s SVC with probability = True); no additional probability calibration was performed, as the Brier score suggested adequate calibration.

Model performance was evaluated by AUC, accuracy, specificity, F1 score, and Brier score, with 95% confidence intervals derived from 1000 bootstrap resamples. Calibration was further assessed using calibration curves, calibration intercept, and calibration slope. Decision curve analysis (DCA) was performed to evaluate clinical utility, with a pre-specified clinically relevant threshold probability range of 10% to 40%.

SHAP (SHapley Additive exPlanations) was used to interpret model predictions and quantify feature contributions. SHAP values were averaged across all patients to assess predictive importance. For one-hot encoded categorical variables, SHAP values were interpreted in terms of relative importance rather than ordinal risk magnitude. SHAP was implemented in Python using version 0.40.0 (Figure 1).

## 3. Results

### 3.1. Patient Characteristics

This study included 671 patients who underwent hypospadias repair at our institution. Among them, 154 patients (23.0%) experienced postoperative complications, while 517 patients (77.0%) did not. There were no statistically significant differences in age (*p* = 0.762) or BMI (*p* = 0.467) between the complication and non-complication groups. The incidence of complications increased with more severe hypospadias types: the proportions of scrotal (37.7%) and perineal (12.3%) types were significantly higher in the complication group than in the non-complication group (14.1% and 3.3%, respectively), whereas the coronal type was more prevalent in the non-complication group (28.0% vs. 8.4%; *p* < 0.001). Complication rates also differed significantly across surgical techniques (*p* < 0.001). TIP was the most commonly used technique overall (49.9%) and was significantly more prevalent in the non-complication group (55.1%) than in the complication group (32.5%). Notably, two-stage repair accounted for 39.0% of cases in the complication group, compared with only 6.4% in the non-complication group. Regarding surgeon volume, the low-volume group (≤200 cases) accounted for 71.4% of the complication group, whereas the high-volume group (>200 cases) accounted for 28.6% (*p* < 0.001), suggesting that lower surgeon volume was associated with higher complication risk. No statistically significant difference was observed in the distribution of surgical periods (early: 2015–2019 vs. recent: 2020–2024) between the two groups (*p* = 0.418) (Table 1).

### 3.2. Follow-Up Outcomes

A total of 671 patients were included in the final analysis, with follow-up censored in April 2026. The median follow-up duration was 48 months (range: 19 to 72 months). All patients completed at least 12 months of follow-up (671/671, 100%), of whom 606 (90.3%) completed ≥ 24 months and 515 (76.8%) completed ≥ 36 months. Postoperative complications occurred in 154 patients (22.9%), including urethral fistula (117 cases, 17.4%), urethral stricture (43 cases, 6.4%), urethral diverticulum (18 cases, 2.7%), and surgical-site infection (2 cases, 0.3%). Complications were counted at the patient level. Since 32 patients (4.8%) experienced two or more types of complications simultaneously, the sum of individual complication counts (180 events) exceeded the total number of patients with complications (154 cases) (Table 2).

As shown in Table 2, 24.7% of patients with postoperative complications presented their first adverse event beyond the standardized 12-month follow-up period, confirming that our long-term follow-up strategy with a median duration of 48 months effectively captured a large number of delayed urethral fistulas and strictures that would be missed by short-term 12-month-only surveillance. Nevertheless, all follow-up data were censored at 72 months, and complications emerging more than 6 years after surgery were unavailable for analysis, which remains an inherent limitation of this retrospective cohort.

### 3.3. Feature Selection

**Figure 2:**
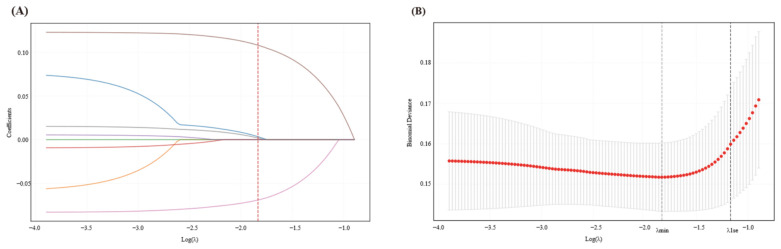
LASSO regression feature selection results for variables in the training dataset. (**A**) LASSO regression coefficient profiles of variables in the training dataset. Different colored lines represent the coefficient trajectories of individual variables as the penalty parameter λ increases. (**B**) Selection of the optimal parameter (λ) in the LASSO regression. The two vertical dashed lines indicate the λ values selected by 10-fold cross-validation: the left dashed line represents λmin (the λ with minimum cross-validation error), and the right dashed line represents λ1se (the largest λ within one standard error of the minimum).

This technique applies L1 regularization to the loss function, compressing coefficients of low-contribution variables to zero, thereby selecting a parsimonious set of predictors. Based on this approach, we ultimately retained four features for model development: surgical approach, surgeon case-volume group (high-volume versus low-volume), hypospadias type, and patient age. To ensure fair comparisons under identical input conditions, all subsequent ML models were uniformly trained and validated using this feature set (Figure 2).

### 3.4. Comparative Analysis of Model Performance

**Figure 3:**
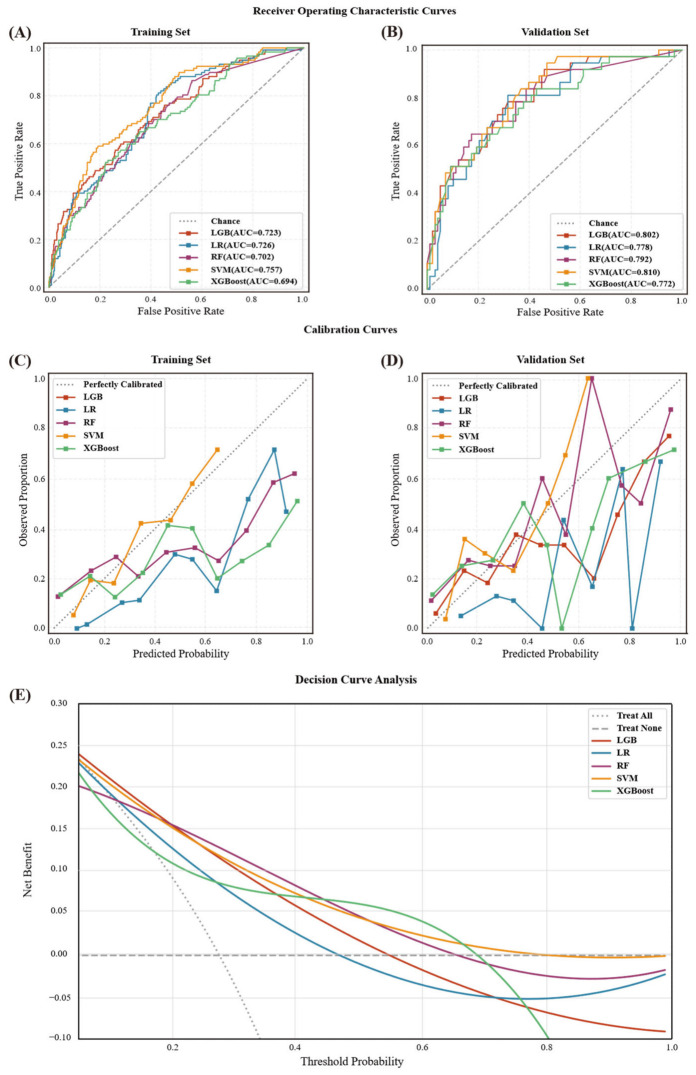
Performance comparison of five models. (**A**) Receiver Operating Characteristic Curves in the training set. (**B**) Receiver Operating Characteristic Curves in the validation set. (**C**) Calibration Curve in the training set. (**D**) Calibration Curve in the validation set. (**E**) Decision Curve Analysis in the validation set.

Within the training set, different ML models displayed distinct performance characteristics (Table 3, Figure 3A). The SVM achieved the highest AUC (0.757), while the LR model exhibited high sensitivity (0.752). Among ensemble learning models, LGB achieved the highest F1 score (0.457). In the validation set, model generalization performance was further evaluated (Table 4, Figure 3B). The SVM achieved the highest AUC (0.810). The RF model achieved the highest accuracy (0.778) and specificity (0.878). Notably, LGB and SVM showed identical specificity (0.765) and F1 score (0.571) in the validation set, with SVM achieving a marginally higher AUC (0.810 vs. 0.802). The LR model had the lowest specificity in the validation set (0.673) among all models. Regarding calibration, the SVM achieved the lowest Brier scores in both the training (0.145) and validation (0.157) sets (Table 5 and Table 6, Figure 3C,D), indicating that its predicted probabilities were closest to the observed outcomes.

The SVM showed higher AUC in the validation set (0.810) than in the training set (0.757). This difference is likely attributable to three factors. First, cross-validation AUC is derived from sub-models trained on 90% of the data and tested on the held-out fold; these “out-of-bag” predictions tend to be conservative, and the aggregated AUC may underestimate the performance of the final model trained on the full training set. Second, the validation set was relatively small (*n* = 135, 35 positive events), making performance metrics subject to sampling variability; the validation AUC (0.810) fell within the 95% bootstrap confidence interval of the cross-validation AUC, confirming that the observed difference was within the expected range of random fluctuation. Third, the improvement in sensitivity from 0.154 in the training set to 0.649 in the validation set reflects the threshold-dependent nature of this metric; the default classification threshold of 0.5 was used for all models. Calibration analysis confirmed that the SVM’s predicted probabilities were well calibrated in the validation set (Table 5 and Table 6), ruling out systematic miscalibration as a contributing factor. The confusion matrix for the SVM model in the validation set at the default classification threshold of 0.5 is provided in Supplementary Figure S1. These findings suggest that SVM is a suitable model in this internal validation, rather than demonstrating its universal superiority.

Decision curve analysis (DCA) was performed to evaluate the clinical utility of the five models across a pre-specified clinically relevant threshold probability range of 10% to 40% (Figure 3E). Within this range, all models provided positive net benefit compared with the “treat none” strategy. The SVM model achieved the highest or tied-highest net benefit across most threshold probabilities, particularly within the 10% to 30% range. At higher threshold probabilities (>40%), the net benefit of all models approached zero. The modest differences in net benefit among the five models suggest that the choice of algorithm may have limited impact on clinical decision-making in this context.

### 3.5. Model Interpretation

**Figure 4:**
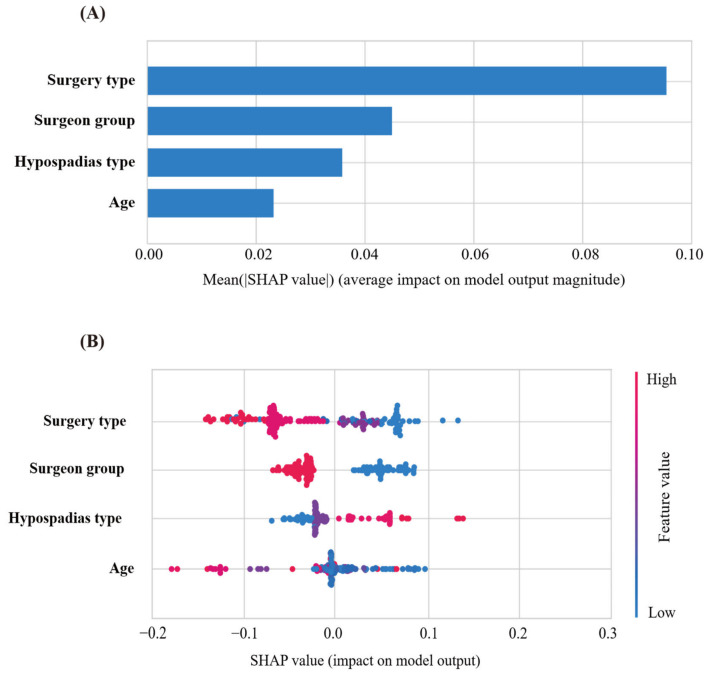
(**A**) SHAP feature importance depicted according to the mean absolute SHAP value of each feature. (**B**) SHAP summary plot indicating the distribution of SHAP values of each feature. Each dot represents a SHAP value for a feature per patient. The x-axis represents the SHAP value, and the color, from red to blue, represents the feature value from high to low.

As shown in Figure 4A, based on the average absolute SHAP values, surgical technique ranked as the most influential predictor for the SVM model, with substantially greater importance than the other variables. Among the surgical technique categories, two-stage repair contributed the highest positive SHAP values, suggesting that the distinction between one-stage and two-stage procedures was a major driver of the model’s predictions. Within one-stage techniques, Duckett and TIP showed variable effects, but their contributions were smaller in magnitude than that of two-stage repair. Surgeon volume and hypospadias type ranked next in importance, whereas age had a relatively minor influence.

The SHAP summary plot in Figure 4B further illustrates the direction and magnitude of each feature’s contribution to the predicted risk. For surgical techniques, the two-stage repair category was predominantly associated with positive SHAP values, indicating an increased predicted risk, whereas other techniques showed more variable contributions. Regarding surgeon volume, the low-volume group exhibited a stronger association with positive SHAP values, suggesting that low surgeon volume was associated with higher predicted risk in the model. For hypospadias type, more severe forms (perineal type) were predominantly associated with positive SHAP values, while milder forms (coronal type) were concentrated in the region of negative SHAP values, indicating that greater anatomical severity was associated with higher predicted complication risk.

Overall, the model assessed risk based on two modifiable procedural factors—surgical technique and surgeon volume—alongside the non-modifiable disease-specific factor of hypospadias type. These findings are consistent with established clinical knowledge and provide a clinically plausible explanation for the model’s predictions. Specifically, undergoing a two-stage repair, being operated on by a low-volume surgeon, and having a more severe hypospadias type were the main contributors to the model’s classification of higher patient risk.

## 4. Discussion

Artificial intelligence has begun to enter hypospadias research, mainly for objective measurement of anatomical features and automated classification. Deep learning has been applied to quantify penile curvature [16,17], assess urethral plate morphology [18], and support diagnostic pattern recognition [19]. Other groups have used machine learning to correlate anogenital distance with hypospadias severity [20] or to explore genotype–phenotype associations [21]. These efforts confirm the potential of AI to reduce subjectivity in pediatric urology. However, few have tackled the question of how to convert routine clinical data into prospective, individualized predictions of surgical complications.

Several nomogram-based prediction models for hypospadias complications have been published in recent years. Mao et al. developed a nomogram incorporating six predictors with internal validation in 553 patients (AUC: 0.821) [8]. Fang et al. conducted a multicenter study of 493 TIP patients and identified anatomical predictors (AUC: 0.723) [9]. Dong et al. reported a nomogram with an AUC of 0.909 for perididymis coverage [10]. Compared with these models, our SVM-based approach achieved a comparable or higher AUC (0.810) using fewer and more readily available clinical variables, suggesting that machine learning may offer advantages in capturing complex interactions among predictors. However, direct comparisons should be interpreted with caution given differences in study populations, outcome definitions, and validation approaches. Notably, the complication rate in our cohort (22.9%) was nearly identical to that reported in the multicenter TIP study (23.7%) [9], supporting the representativeness of our data.

To address this gap, we compared several ML algorithms in a cohort of 671 patients. SVM achieved the most favorable balance between discrimination and calibration: a validation AUC of 0.810 and a Brier score of 0.157. The model performed well not only in ranking risk but also in producing probability estimates that matched observed event rates. This property is especially important for clinical decision-making, where probability estimates—not just rankings—guide communication and planning. RF showed strong training-set performance but diminished in validation, suggesting overfitting. Other ensemble methods fell between these extremes.

The SHAP analysis provided a transparent view of the SVM’s decision logic. Surgical technique had the largest influence on predictions, followed by surgeon volume and hypospadias type. These results align with prior studies showing higher complication rates in proximal cases, complex repairs, and low-volume operators [22,23]. Age contributed only marginally. The American Academy of Pediatrics recommends surgery between 6 and 12 months of age [24], while the European Association of Urology/European Society for Paediatric Urology guidelines recommend repair between 6 and 18 months [25]. The rationale for these recommendations has been primarily based on anesthetic safety, tissue healing, and psychosocial considerations, rather than on complication risk per se. Our model’s modest weighting of age supports the view that age is not a dominant predictor of short-term surgical outcomes. The SHAP-derived ranking was also consistent with baseline univariate comparisons, lending additional credibility to the model’s attributions.

We recognize that the individual predictors themselves are not new. Their prognostic value has been documented repeatedly. The contribution of this work is instead methodological: demonstrating that a machine learning pipeline using routine variables can produce well-calibrated, interpretable risk estimates with performance comparable to or better than published nomograms. This approach could be extended in future work to include intraoperative details—suture choice, chordee correction, and flap vascularity—that are difficult to incorporate into conventional regression models.

From a clinical standpoint, the model can support shared decision-making before surgery. Estimated probabilities can inform discussions about operative strategy, surgeon selection, and postoperative surveillance intensity. After surgery, the same probabilities can guide follow-up scheduling, concentrating resources on patients at highest risk.

Several limitations should be acknowledged. First, the data come from a single institution and are retrospective, which limits generalizability. The risk of overfitting was partially mitigated by nested cross-validation for feature selection and bootstrap resampling for performance evaluation; however, the observed discrepancy between training and validation performance in some models warrants cautious interpretation. Second, we did not stratify by hypospadias location (distal vs. proximal) in the primary analysis due to the limited number of proximal cases (*n* = 167), which would have reduced statistical power and may obscure important subgroup differences. Third, a sensitivity analysis focusing specifically on fistula and stricture was not performed due to the limited number of events for subgroup analysis, although the frequency of each complication type has been provided separately in Table 2. Fourth, some key anatomical predictors—glans width, urethral plate quality, and chordee severity—were not available because they were not part of the routine electronic record during the earlier study years. The PREDICT-H study, an ongoing prospective multicenter effort, is designed to capture such variables systematically and will help address this issue [26]. Fifth, although a web-based calculator or nomogram was not developed in the present study, the model can be readily translated into such tools once external validation is completed. Finally, the 12-month structured follow-up may have missed complications that present later; 24.7% of patients with complications experienced their initial adverse event after the 12-month routine surveillance window. However, the median follow-up of 48 months captured some late events. The study period also spans nearly a decade, during which surgical techniques and materials evolved, potentially introducing temporal bias.

This report follows the TRIPOD + AI checklist for prediction model studies [27]. Future priorities include external validation in independent datasets, incorporation of additional data types (imaging, surgical video, biomarkers), and evaluation of whether model-guided interventions actually improve patient outcomes. Longitudinal outcomes beyond the early postoperative period, including patient-reported satisfaction, should also be integrated into future model iterations.

In summary, this retrospective study demonstrates that a machine learning approach using SVM and routine clinical variables can predict hypospadias repair complications with promising internal validity. The model’s performance and interpretability support further testing in prospective, multicenter settings. With external validation, such tools could eventually inform preoperative counseling and postoperative surveillance, although they are not yet ready for routine clinical deployment.

## 5. Conclusions

An interpretable SVM-based model was developed and internally validated for predicting complications after hypospadias repair. The model performed well in both discrimination and calibration (validation AUC 0.810, Brier 0.157). SHAP analysis identified surgical technique, surgeon volume, and hypospadias type as the main contributors to predicted risk. These results support the feasibility of machine learning for risk stratification using routine clinical data, but external validation is essential before clinical adoption.

## Figures and Tables

**Table 1:** Baseline characteristics comparison between patients with and without complications.

Variable	Total (*n* = 671)	No Complication (*n* = 517)	Complication (*n* = 154)	*p*-Value
Patient characteristics				
Age (years)	2.42 (1.58, 4.00)	2.42 (1.58, 4.00)	2.33 (1.67, 3.21)	0.762
BMI	16.36 (15.15, 17.78)	16.36 (15.04, 17.81)	16.35 (15.41, 17.61)	0.467
Hypospadias type				<0.001
Coronal	158 (23.5)	145 (28.0)	13 (8.4)	
Penile	346 (51.6)	282 (54.5)	64 (41.6)	
Scrotal	131 (19.5)	73 (14.1)	58 (37.7)	
Perineal	36 (5.4)	17 (3.3)	19 (12.3)	
Surgery type				<0.001
Mathieu	19 (2.8)	14 (2.7)	5 (3.2)	
Duckett	77 (11.5)	54 (10.4)	23 (14.9)	
Duckett and Duplay	18 (2.7)	10 (1.9)	8 (5.2)	
Koyanagi	5 (0.7)	4 (0.8)	1 (0.6)	
MAGPI	112 (16.7)	108 (20.9)	4 (2.6)	
Onlay	12 (1.8)	9 (1.7)	3 (1.9)	
TIP	335 (49.9)	285 (55.1)	50 (32.5)	
Two-Stage Repair	93 (13.9)	33 (6.4)	60 (39.0)	
Surgeon group				<0.001
Low-volume (Cases ≤ 200)	336 (50.1)	226 (43.7)	110 (71.4)	
High-volume (Cases > 200)	335 (49.9)	291 (56.3)	44 (28.6)	
Year of surgery				0.418
Early (2015–2019)	457 (68.1)	348 (67.3)	109 (70.8)	
Recent (2020–2024)	214 (31.9)	169 (32.7)	45 (29.2)	

Note: BMI (body mass index), MAGPI (Meatal Advancement and Glanuloplasty Incorporated), Mathieu (perimeatal-based flap urethroplasty), TIP (tubularized incised plate urethroplasty), Onlay (onlay island flap urethroplasty), Duckett (transverse preputial island flap urethroplasty), Koyanagi (parameatal-based flap urethroplasty). Continuous variables with normal distribution are compared using *t*-tests and reported as mean ± standard deviation. Non-normally distributed continuous variables were compared using the Mann–Whitney U test and reported as median (IQR). Categorical variables are expressed as percentages and compared using Pearson’s chi-square (χ^2^) test; categorical variables are presented as counts and percentages. The complication rate for each surgical technique can be calculated from the numbers provided (e.g., TIP: 50/335 = 14.9% complication rate).

**Table 2:** Follow-up and complication profile of the study cohort (*N* = 671).

Category	Characteristic	Value
Follow-up duration	Median follow-up, months (range)	48 (19, 72)
	Total patients, *N*	671
Follow-up completion	≥12 months, *n* (%)	671 (100)
	≥24 months, *n* (%)	606 (90.3)
	≥36 months, *n* (%)	515 (76.8)
Complication profile	Overall complications, *n* (%)	154 (22.9)
	Urethrocutaneous fistula, *n* (%)	117 (17.4)
	Urethral stricture, *n* (%)	43 (6.4)
	Urethral diverticulum-like dilation, *n* (%)	18 (2.7)
	Surgical-site infection, *n* (%)	2 (0.3)
Multiple concurrent complications	Patients with ≥2 types of complications, *n* (%)	32 (4.8)
Onset timing of first complication	Initial event ≤ 12 months, *n* (%)	116 (75.3)
	Initial event > 12 months, *n* (%)	38 (24.7)

Note: Continuous variables are presented as median (range) due to non-normal distribution. Categorical variables are expressed as counts and percentages. Complications were counted at the patient level; therefore, the sum of individual complication counts (180 cases) exceeds the total number of patients with complications (154 cases) due to overlapping events. Patients were stratified by the timing of their first postoperative complication (≤12 months vs. >12 months).

**Table 3:** ROC performance metrics of different ML models in the training set.

Model	AUC (95% CI)	Accuracy (95% CI)	Specificity (95% CI)	F1 Score (95% CI)
LGB	0.723 (0.680–0.766)	0.703 (0.664–0.742)	0.740 (0.698–0.782)	0.457 (0.390–0.524)
LR	0.726 (0.683–0.769)	0.636 (0.596–0.676)	0.604 (0.557–0.651)	0.474 (0.407–0.541)
RF	0.702 (0.657–0.747)	0.733 (0.696–0.770)	0.821 (0.784–0.858)	0.407 (0.340–0.474)
SVM	0.757 (0.715–0.799)	0.793 (0.759–0.827)	0.971 (0.956–0.986)	0.245 (0.162–0.328)
XGBoost	0.694 (0.649–0.739)	0.716 (0.678–0.754)	0.785 (0.746–0.824)	0.420 (0.353–0.487)

Note: Data are presented as point estimate (95% confidence interval). 95% confidence intervals were derived from 1000 bootstrap resamples. AUC, Area Under the Receiver Operating Characteristic curve; LR, Logistic Regression; RF, Random Forest; SVM, Support Vector Machine; LGB, Lightweight Gradient Boosting; XGBoost, Extreme Gradient Boosting.

**Table 4:** ROC performance metrics of different ML models in the validation set.

Model	AUC (95% CI)	Accuracy (95% CI)	Specificity (95% CI)	F1 Score (95% CI)
LGB	0.802 (0.735–0.869)	0.733 (0.659–0.807)	0.765 (0.688–0.842)	0.571 (0.470–0.672)
LR	0.778 (0.707–0.849)	0.711 (0.635–0.787)	0.673 (0.591–0.755)	0.606 (0.506–0.706)
RF	0.792 (0.722–0.862)	0.778 (0.707–0.849)	0.878 (0.814–0.942)	0.559 (0.456–0.662)
SVM	0.810 (0.743–0.877)	0.733 (0.659–0.807)	0.765 (0.688–0.842)	0.571 (0.470–0.672)
XGBoost	0.772 (0.699–0.845)	0.763 (0.691–0.835)	0.857 (0.790–0.924)	0.543 (0.440–0.646)

Note: Data are presented as point estimate (95% confidence interval). 95% confidence intervals were derived from 1000 bootstrap resamples. AUC, Area Under the Receiver Operating Characteristic curve; LR, Logistic Regression; RF, Random Forest; SVM, Support Vector Machine; LGB, Lightweight Gradient Boosting; XGBoost, Extreme Gradient Boosting.

**Table 5:** Calibration metrics of different ML models in the training set.

Model	Brier Score (95% CI)	Calibration Intercept	Calibration Slope
LGB	0.198 (0.178–0.218)	−1.256	0.877
LR	0.205 (0.185–0.225)	−0.856	0.079
RF	0.188 (0.169–0.207)	0.096	1.078
SVM	0.145 (0.127–0.163)	−0.979	0.414
XGBoost	0.212 (0.191–0.233)	−0.919	0.247

Note: Data are presented as point estimate (95% confidence interval) for Brier score, and as point estimate for calibration intercept and calibration slope. Brier score ranges from 0 to 1, with lower values indicating better calibration. The calibration intercept and slope reflect calibration-in-the-large and calibration slope, respectively, with ideal values of 0 and 1. 95% confidence intervals for Brier score were derived from 1000 bootstrap resamples. LR, Logistic Regression; RF, Random Forest; SVM, Support Vector Machine; LGB, Lightweight Gradient Boosting; XGBoost, Extreme Gradient Boosting.

**Table 6:** Calibration metrics of different ML models in the validation set.

Model	Brier Score (95% CI)	Calibration Intercept	Calibration Slope
LGB	0.171 (0.131–0.211)	−0.914	1.000
LR	0.190 (0.148–0.232)	−0.437	0.120
RF	0.164 (0.126–0.202)	0.428	1.085
SVM	0.157 (0.121–0.193)	−0.639	0.552
XGBoost	0.181 (0.141–0.221)	−0.421	0.335

Note: Data are presented as point estimate (95% confidence interval) for Brier score, and as point estimate for calibration intercept and calibration slope. Brier score ranges from 0 to 1, with lower values indicating better calibration. The calibration intercept and slope reflect calibration-in-the-large and calibration slope, respectively, with ideal values of 0 and 1. 95% confidence intervals for Brier score were derived from 1000 bootstrap resamples. LR, Logistic Regression; RF, Random Forest; SVM, Support Vector Machine; LGB, Lightweight Gradient Boosting; XGBoost, Extreme Gradient Boosting.

## Data Availability

The data presented in this study are available on request from the corresponding author to ensure patient privacy.

## Supplementary Materials

The following supporting information can be downloaded at: https://www.mdpi.com/article/10.3390/children13070962/s1. Supplementary Figure S1: Confusion matrices for the SVM model in the training set (cross-validation) and the validation set at the default classification threshold of 0.5 (scikit-learn predict default). Left: training set (10-fold cross-validation); Right: validation set.
