# Supplementary material for: Machine Learning for Predicting Postoperative Complications After Hypospadias Surgery: A 10-Year Single-Center Retrospective Cohort Study
Source: Children (Basel). 2026 Jul 21;13(7):962. doi: 10.3390/children13070962 (PMC13407137; doi:10.3390/children13070962)
Supplement: Supplementary file 1 [file children-13-00962-s001.zip › children-4362085-supplementary.pdf]

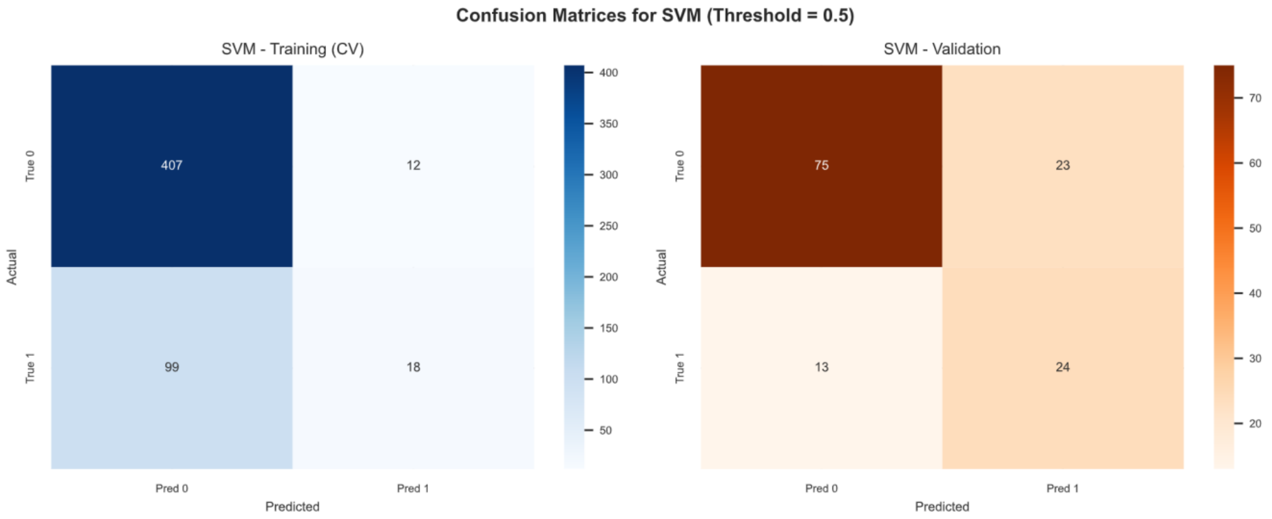

Supplementary Figure S1. Confusion matrices for the SVM model in the training set (cross-validation) and the validation set at the default classification threshold of 0.5 (scikit-learn predict() default). Left: training set (10-fold cross-validation); Right: validation set.
